# Case Report: Synchronous minute intramucosal Epstein–Barr virus-associated gastric cancer revealed after short-term *Helicobacter pylori* eradication

**DOI:** 10.3389/fmed.2026.1846160

**Published:** 2026-06-30

**Authors:** Dongqi Wu, Quan Luo, Weiming Wang, Xueman Wang

**Affiliations:** Endoscopy Center, Shaoxing People's Hospital, The First Affiliated Hospital, Shaoxing University, Shaoxing, China

**Keywords:** early gastric cancer, EBV-associated gastric cancer, endoscopic submucosal dissection, gastric carcinoma with lymphoid stroma, *Helicobacter pylori*, lace pattern

## Abstract

Epstein–Barr virus-associated gastric cancer (EBVaGC) is a rare subtype, accounting for approximately 8.7% of gastric cancers worldwide. It predominantly affects males and typically presents endoscopically as a superficial depressed lesion or with a submucosal tumor-like appearance. Pathologically, it is characterized by prominent lymphoid stromal infiltration (gastric carcinoma with lymphoid stroma, GCLS). Molecular features include extensive CpG island methylation, PIK3CA mutations, and high PD-L1/PD-L2 expression. We report the case of a 56-year-old patient diagnosed with gastric adenocarcinoma at an outside institution. Upon admission, magnifying endoscopy with narrow-band imaging (ME-NBI) revealed active *Helicobacter pylori* (HP) infection and a Paris 0-IIc reddish lesion on the posterior wall of the middle gastric body with indistinct margins. The patient underwent a 2-week HP eradication regimen. Subsequent endoscopic submucosal dissection (ESD) revealed two distinct superficial depressed lesions, which were removed en bloc. The lesions measured 15 mm (distal lesion) and 26 mm (proximal lesion), respectively. Postoperative pathology confirmed two independent foci of intramucosal EBVaGC with positive EBER *in situ* hybridization. The lesions were limited to the mucosa (pTis) without lymphovascular invasion, achieving curative resection (eCura A). At the 6-month follow-up, no recurrence was observed. This case suggests that in the setting of active HP-associated gastritis, EBVaGC may be obscured; Short-term preoperative HP eradication may alleviate background inflammation, potentially improving the detection of minute lesions and the delineation of margins. These findings form a hypothesis that eradication might aid endoscopic assessment, which requires validation in larger cohorts. Under strict risk assessment and margin control, ESD represents a curative modality for early EBVaGC.

## Introduction

Epstein–Barr virus (EBV) is a herpesvirus associated with various human malignancies, including nasopharyngeal carcinoma, lymphoma, and EBV-associated gastric cancer (EBVaGC) (1). EBVaGC was first identified by Burke et al. (35) via PCR in undifferentiated gastric carcinomas with intense lymphoid infiltration and is currently recognized by TCGA as one of the four major molecular subtypes of gastric cancer, accounting for approximately 7.5%−10.0% of global cases (2, 3). Its molecular profile is distinct, featuring extensive genomic CpG island methylation, PIK3CA mutations, and overexpression of PD-L1/PD-L2 (4). Clinically, EBVaGC shows a male predominance and a predilection for the proximal gastric body or remnant stomach. Endoscopically, it often manifests as a superficial depressed lesion or a submucosal tumor-like mass. Histopathologically, the presence of massive lymphoid infiltration defines it as gastric carcinoma with lymphoid stroma (GCLS) (5, 6), characterized by anastomosing neoplastic cords embedded in dense lymphocytes—the so-called “lace pattern” (7, 8). Definitive diagnosis relies on demonstration of strong nuclear EBER expression by *in situ* hybridization. Accumulating clinicopathological data indicate that EBVaGC is less aggressive than EBV-negative early gastric cancer (5, 9). Although some authors (10, 11) have proposed incorporating EBV positivity with lymphovascular invasion-negative status as a favorable factor for expanding endoscopic submucosal dissection (ESD) cure criteria, this has not yet been formally adopted in current guidelines (9). In this case, EBV status served solely as a favorable prognostic indicator, rather than a basis for therapeutic decision-making. Active *Helicobacter pylori* (HP) gastritis, marked by diffuse mucosal erythema, edema, and enlarged gastric folds, can obscure minute neoplastic lesions and blur demarcation lines. While no current clinical guidelines recommend routine pre-ESD HP eradication, previous work by Nakagawa et al. demonstrated that successful HP eradication may induce partial morphologic changes of early gastric cancer, clarify lesion borders, and unmask previously occult synchronous minute foci, thereby facilitating precise margin marking for ESD (12). We report a case of synchronous intramucosal EBVaGC (GCLS type) presenting as two independent lesions, successfully cured via ESD following HP eradication. We discuss the diagnostic criteria, therapeutic decision-making, and biological significance of this case in the context of relevant literature.

## Case description

**Figure 1:**
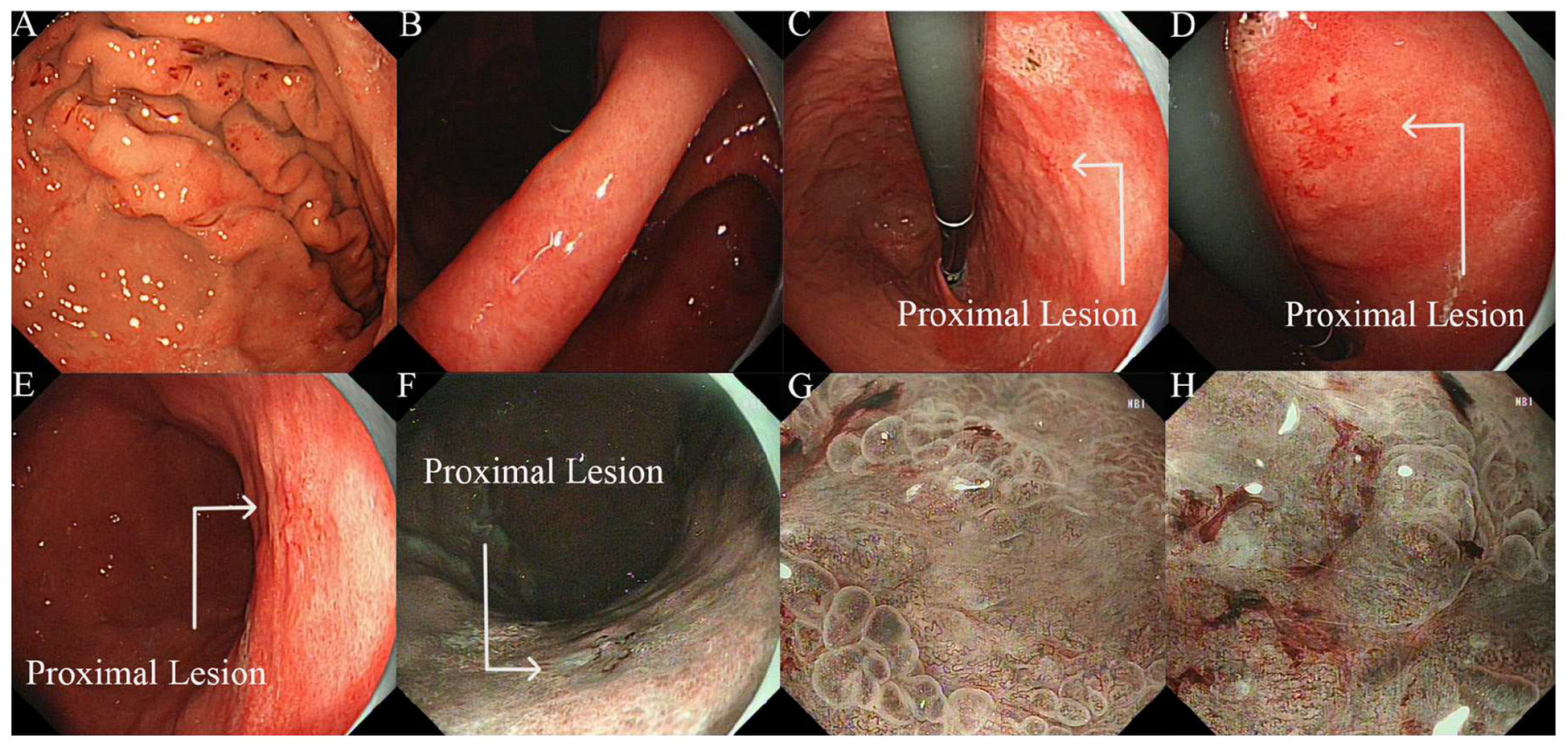
**(A, B)** Active *helicobacter pylori* infection: diffuse redness, mucosal swelling, enlarged fold. **(C–F)** White light imaging (WLI) and narrow-band imaging (NBI): a Paris type 0-IIc lesion without clear demarcation. **(G, H)** Magnifying endoscopy with narrow-band imaging (ME-NBI): irregular microvascular pattern (IMVP+), irregular microsurface pattern (IMSP+), the demarcation line (DL) is ill-defined.

A 56-year-old patient presented to our department in June 2025, 2 weeks after an external medical checkup revealed gastric adenocarcinoma. The patient was asymptomatic and had no positive physical signs upon admission. His medical history included surgery for pulmonary mucinous adenocarcinoma in August 2024. Personal and family histories were non-contributory. Physical examination upon admission was unremarkable. Admission laboratory tests (tumor markers) and contrast-enhanced abdominal CT were unremarkable. The initial gastroscopy at the outside facility (May 20, 2025) reported “erosive gastritis.” Biopsy of the posterior wall of the middle gastric body indicated mucosal erosive inflammation with disordered atypical glands, favoring adenocarcinoma. HP testing was positive. To determine the optimal resection strategy, we performed magnifying endoscopy with narrow-band imaging (ME-NBI; Figure 1).

### White light imaging (WLI)

Diffuse redness, mucosal swelling, enlarged fold, and loss of regular arrangement of collecting venules were observed, consistent with active HP infection. A suspicious Paris type 0-IIc lesion was identified on the posterior wall of the middle gastric body, presenting as mild mucosal redness with fine erosive changes. There was no significant convergence of folds. The area of discoloration measured approximately 1.5 cm; however, the background active inflammation prevented accurate assessment of the lesion's size, boundaries, and degree of differentiation (Figure 1).

### ME-NBI findings

A demarcation line (DL) was suspected but indistinct due to background inflammation. Within the lesion, the microvascular pattern was irregular, dilated, and dense (Irregular Microvascular Pattern, IMVP+). The microsurface pattern was blurred or partially absent (Irregular Microsurface Pattern, IMSP+), suggesting an undifferentiated component. No “non-extension sign” or other indicators of deep submucosal invasion were noted. Based on the 7th edition of the Japanese Gastric Cancer Association guidelines and the eCura assessment framework, the lesion was preoperatively staged as cT1a, ≤ 2 cm, without ulceration, fitting the indications for undifferentiated-type early gastric cancer. We planned for curative ESD. Given the interference of HP-induced inflammation with margin assessment, we opted for prior HP eradication (Figure 1).

### The HP eradication

The patient received Vonoprazan (20 mg bid) and Amoxicillin (1 g tid) for 14 days. Adherence was good, with no adverse events. As ESD was scheduled shortly after eradication, a 13C-urea breath test was planned for ≥4 weeks post-treatment to confirm eradication status.

**Figure 2:**
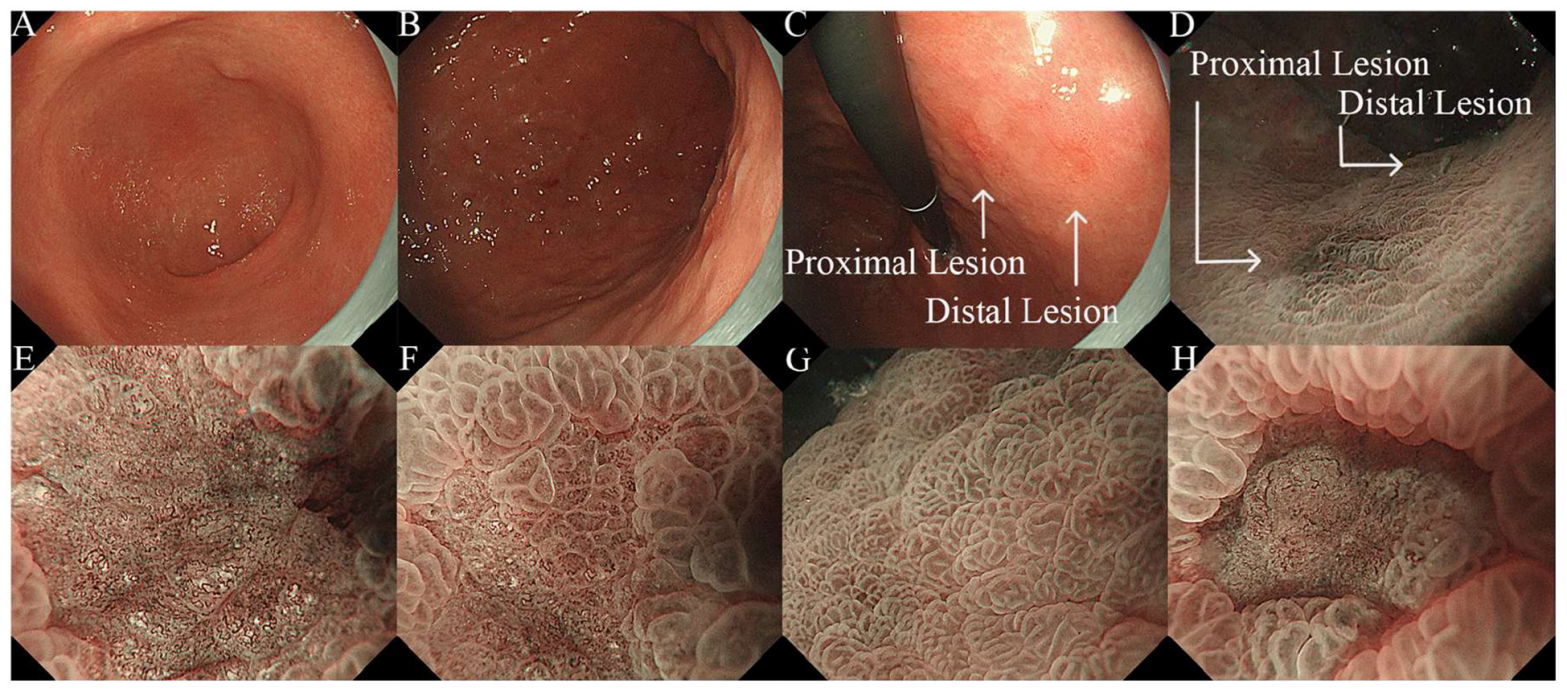
**(A, B)** The inflammation has improved: the diffuse redness has subsided. Mucosal swelling has decreased. The enlarged folds have improved. **(C, D)** WLI and NBI: two separate lesions with distinct demarcation line. **(E, F)** Proximal lesion on ME-NBI: endoscopic lace pattern (ELP). **(G)** The intervening normal tissue on ME-NBI: between them, an intact normal microstructure and a regular microvascular network are observed. **(H)** Distal lesion on ME-NBI:ELP.

Two weeks after completing eradication, ME-NBI was repeated (Figure 2). On white light, mucosal inflammation had significantly subsided. Two distinct Paris 0-IIc reddish lesions (approximately 2.5 cm and 1.5 cm) were now visible on the posterior wall of the middle gastric body. Proximal lesion: distinct demarcation line (DL+). Irregular, dilated, tea-colored microvessels (IMVP+). Fused/absent crypts (IMSP+) with a white globe appearance (WGA). Distal lesion: similar appearance, with more pronounced vascular heterogeneity and tortuosity (IMVP+). Fused glands in the depressed area (IMSP+). Both lesions exhibited irregular/absent microsurface structures accompanied by disordered, dense microvessels, forming a fine, dense subepithelial vascular network characteristic of the endoscopic lace pattern (ELP). Normal microsurface structure and regular microvasculature were observed in the gap between the lesions (approximately 8 mm), consistent with the postoperative pathology reconstruction which confirmed non-neoplastic tissue in the inter-lesional zone. The proximal lesion showed clearer margins after eradication than before, but its size exceeded 2 cm. ME-NBI observation suggested the presence of undifferentiated components. According to the 7th edition of the Japanese Gastric Cancer Association guidelines, this falls under the relative indication for ESD. If postoperative pathology confirms that undifferentiated components are dominant, the curative resection evaluation would be classified as eCura C2, necessitating additional surgical intervention after ESD. The distal lesion, newly detected after eradication, meets the absolute indication for ESD. However, after communication with the family, they requested to proceed with ESD first. Although the lesions appeared independent endoscopically, the distance between them was short; therefore, en bloc ESD was performed.

**Figure 3:**
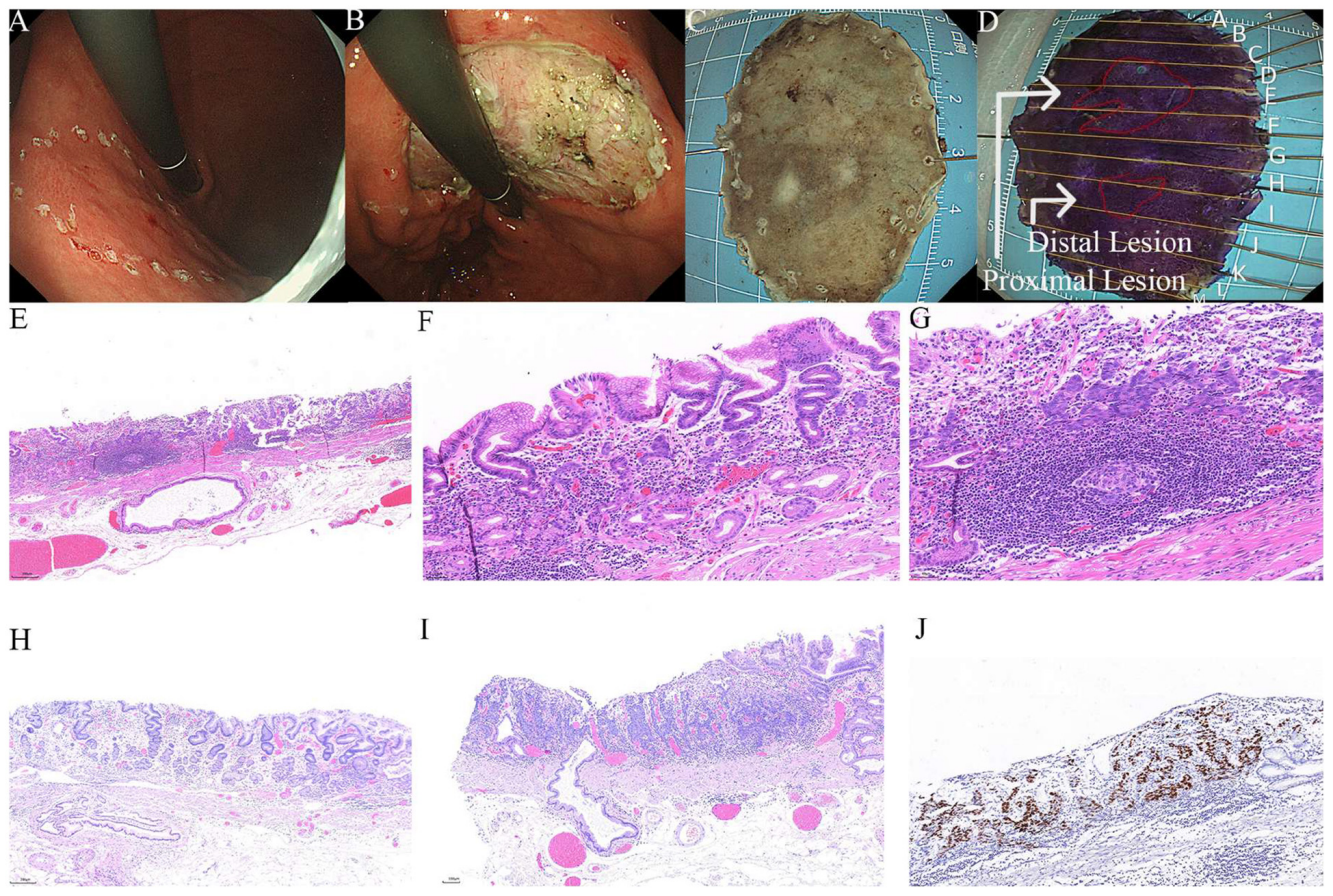
**(A–D)** Surgical procedure. **(A)** Marking the extent of the lesion. **(B)** Postoperative wound. **(C)** Surgical specimen. **(D)** Specimen restoration. **(E–I)** Hematoxylin and eosin (H&E)-stained sections from surgical specimens. **(E)** Proximal lesion: the lesion is well-demarcated with a dense lymphocytic infiltration within the stroma (original magnification, ×100). **(F)** Proximal lesion: at the border, tumor cells are seen beneath the normal foveolar epithelium, horizontally extending into the mid portion of the mucosa (original magnification, ×400). **(G)** Proximal lesion: the lesion demonstrate irregularly anastomosing neoplastic tubules accompanied by moderate to dense lymphocytic infiltration (Lace Pattern) (original magnification, ×400). **(H)** The endoscopically normal-appearing mucosa was confirmed by pathology to be free of tumor extension (original magnification, ×100). **(I)** Distal lesion: lace pattern (original magnification, ×200). **(J)** Immunohistochemical staining: positive for EBER (original magnification, ×400).

ESD was performed under general anesthesia. The lesion margins were marked using a Dual Knife. Following submucosal injection of saline-indigo carmine solution to achieve lifting, a circumferential incision and submucosal dissection were completed. Hemostasis was managed with hemostatic forceps, including prophylactic coagulation of exposed small vessels. En bloc resection was achieved in 60 min without perforation or delayed bleeding (Figure 3).

### Postoperative pathology

Specimen Size (Figure 3): 5.6 × 4.1 cm (referring to the total area encompassing both foci). Tumor location: gastric body. Macroscopic type: superficial depressed (Paris 0-IIc).The maximum diameter of the distal lesion was 15 mm, and the proximal lesion was 26 mm. Inter-lesional distance approx 10 mm (confirmed tumor-free). Background mucosa: chronic moderate atrophic gastritis, mild activity, mild intestinal metaplasia. Histology: moderately to poorly differentiated tubular adenocarcinoma (tub2 > por1). Depth: limited to mucosa (pTis).Features: no ulceration (UL-), no lymphovascular invasion (Ly0, V0). Horizontal and vertical margins negative (HM0, VM0). Immunohistochemistry (IHC): CD10(–), MUC2(–), MUC5ac(–), MUC6(–), P53 (wild type), SMA (muscularis mucosae intact), Desmin (muscularis mucosae intact), Ki67 (+35%).

Pathological reconstruction (Figure 3): the specimen was sectioned into 13 slices. Lesions were located in slices D/E/F and slice I, with slices G/H free of tumor. Histologically, cancer nests exhibited dense infiltration of lymphocytes and plasma cells. In some areas, cancer cells formed irregular, anastomosing small trabeculae or glandular structures embedded within the lymphoid stroma, displaying the typical “Lace Pattern (LP).” EBER *in situ* hybridization confirmed positivity in both lesions.

### Final diagnosis

Synchronous intramucosal EBV-associated gastric cancer (GCLS type), tub2 > por1, pTis, UL(–), Ly0, V0, HM0, VM0, Curative ESD resection (eCura A). Given the extremely low risk of lymph node metastasis in mucosal EBVaGC without lymphovascular invasion, and confirming two independent lesions both meeting the eCura A criteria, the multidisciplinary team (MDT) decided against additional surgery. The patient was placed on a surveillance program with periodic endoscopy and imaging.

**Figure 4:**
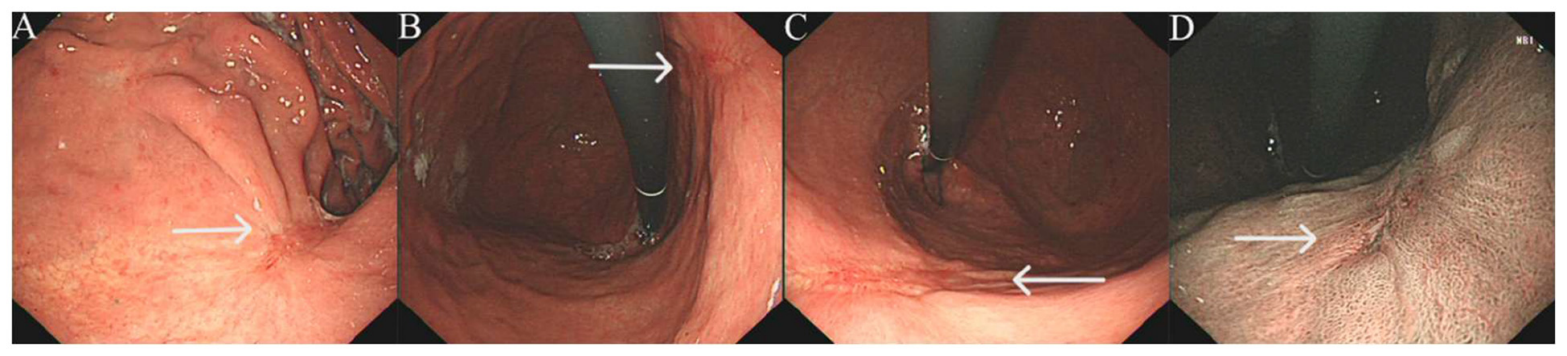
**(A, B)** 3-month follow-up. **(C, D)** 6-month follow-up. The ESD ulcer site has epithelialized, presenting as smooth, slightly erythematous scar tissue without bleeding, erosion, or residual ulceration. The surrounding gastric mucosa shows significant improvement in background inflammation compared to the preoperative state.

The patient underwent follow-up endoscopy at 3 and 6 months post-ESD (Figure 4). Under white light endoscopy, the ulcer surface at the ESD site had largely epithelialized, appearing as smooth, slightly erythematous scar tissue without any evidence of bleeding, erosion, or residual ulceration. The background inflammation of the surrounding gastric mucosa showed marked improvement compared to the preoperative state, aligning with a HP-negative status. Overall, the gastroscopic presentation was consistent with favorable clinical healing, and the patient reported no subjective complaints or discomfort.

Patient timeline is shown in Table 1.

**Table 1:** Clinical timeline.

Time	Event	Key outcome
May 20, 2025	Initial outside workup	Biopsy: adenocarcinoma; Hp+
Jun 02, 2025	Admission evaluation	ME-NBI: active Hp gastritis, 1 ill-defined 0-IIc lesion
Jun 03, 2025	HP eradication	14-day vonoprazan + amoxicillin
Jun 18, 2025 (2 weeks post)	Repeat ME-NBI + ESD	Inflammation resolved; 2 distinct lesions (26 mm/15 mm) with ELP; en bloc resection
Post-ESD day 3	Pathology & MDT	eCura A (pTis,UL-,tub2 > por1, Ly0/V0); MDT advised surveillance over surgery
3/6 months post-ESD	Follow-up	Ulcer healed, no recurrence, or metachronous lesions

## Discussion

The primary insight from this case is the impact of active HP infection on the endoscopic visibility of EBVaGC. Background inflammation can obscure the demarcation line and extent of the lesion, increasing the risk of missed diagnosis or positive margins (6, 13, 14). Initially, only one indefinite lesion was identified. Following short-term HP eradication, reduced inflammation unmasked two independent lesions and the intervening normal mucosa, allowing for a single en bloc ESD and minimizing the risk of missing synchronous micro-lesions. Although causality cannot be claimed, this observation suggests that short-term HP eradication may benefit patients whose lesions are obscured by active gastritis.

EBVaGC is a distinct subtype characterized by unique molecular and immune microenvironmental features (15). Latent EBV infection induces epigenetic and signaling pathway alterations in the host, often accompanied by a “lymphocyte-rich” tumor microenvironment (the GCLS phenotype) (16). HP and EBV may act independently or synergistically in gastric carcinogenesis (3, 17). Studies (18) suggest co-infection significantly increases the risk of precancerous lesions and intestinal-type gastric cancer (19). HP damages the mucosal barrier, enhancing EBV infection efficiency, while amplification of inflammation, epigenetic changes, and signaling crosstalk promote tumor progression (3, 17, 20). While this synergy may make EBVaGC more aggressive in an inflammatory setting, the associated immune infiltration may limit metastasis (5, 21). In some cases, HP-related inflammation superimposes on the EBV-associated immune response, making early endoscopic recognition difficult. In our case, the “unmasking” of the lesions post-eradication suggests that once inflammatory interference is reduced, the structural and vascular abnormalities specific to EBV-associated tumors (such as ELP) are more readily captured by ME-NBI. However, this inference still requires more forward-looking research for verification (22).

The “Lace Pattern,” first described by Uemura et al. (23). in 1994, is a histological feature of intramucosal EBVaGC, showing branching and anastomosing tubules with marked lymphocytic infiltration within the carcinoma nests. The endoscopic lace pattern (24) seen on ME-NBI—defined as loss or obscuration of the microsurface pattern with a dense, irregular subepithelial capillary network—corresponds to this pathological finding. Accurate identification of EBVaGC relies on integrating endoscopic and pathological findings. In this case, the lesions was Paris 0-IIc type in the middle gastric body. ME-NBI revealed an irregular/absent microsurface and disordered, dense microvessels forming a fine subepithelial network—the ELP. Postoperative pathology confirmed neoplastic tubules forming irregular anastomosing structures surrounded by lymphocytes (histological LP), correlating with the ELP (6). EBER positivity provided definitive confirmation (25). This case shows how the diagnosis came together: we first spotted the ELP, leading to a suspicion of EBVaGC, which was then validated by characteristic histological features(LP) and confirmed by molecular evidence (EBER). Clinicians encountering proximal superficial depressed lesions with ELP on ME-NBI should maintain a high index of suspicion for EBVaGC and request EBER testing.

Regarding treatment, the MDT recommended surveillance rather than additional surgery based on two factors. Pathological criteria: the lesions were independent, tub2 > por1, intramucosal, non-ulcerated, lymphovascular invasion (LVI)-negative, and resection margins were negative (eCura A), indicating a negligible risk of lymph node metastasis (26, 27). Biological behavior: research by Osumi et al. (9) indicates that pT1a EBVaGC has an extremely low lymph node metastasis rate, likely due to the antitumor immune microenvironment. Although Osumi et al. (9) proposed that pT1a EBVaGC carries a near-zero risk of lymph node metastasis based on large retrospective cohorts, advocating for its inclusion in expanded ESD indications, these findings require cautious interpretation. Most supportive data derive from radical gastrectomy specimens, whereas long-term post-ESD surveillance data remain scarce. Similarly, Park et al. (11) and Lim et al. (10) suggest that even in SM2 (< 2000 μm) EBVaGC, the risk of metastasis is very low in the absence of lymphovascular invasion. Kobayashi et al.'s (7) summary of pT1b GCLS-type EBVaGC found no nodal metastasis in patients who underwent additional surgery, yet the sample size was limited and cases with LVI were largely excluded. While EBV positivity alone does not supersede standard curative criteria—and this approach has not been formally adopted in current guidelines—EBVaGC (GCLS type) generally exhibits indolent behavior when meeting criteria such as mucosal confinement and R0 resection. Conversely, standard indications for additional surgery remain applicable in the presence of submucosal invasion, LVI, or positive margins. In this case, EBV status served solely as a favorable prognostic indicator rather than a basis for therapeutic decision-making.

Regarding the timing of eradication: While the long-term benefit of HP eradication lies in preventing metachronous cancer, this case highlights a specific short-term clinical utility (28). In scenarios where active inflammation compromises margin delineation, pre-ESD eradication may improve lesion detection and margin assessment (29). However, clinicians must be aware of post-eradication phenomena such as “map-like redness” (where atrophic areas appear redder than non-atrophic mucosa) (30, 31), potential adverse drug reactions, and the risk of treatment delay (32). Regarding the impact of HP eradication, existing literature primarily focuses on long-term chemoprevention, while its short-term diagnostic value remains hypothetical. Nakagawa et al. (12) documented morphological regression of early gastric cancer following eradication; however, whether such regression exhibits unique features in EBVaGC remains unclear. The distinct immune microenvironment of EBVaGC may render it particularly sensitive to inflammatory fluctuations following eradication (33). Notably, Noh et al. (34) reported differential enrichment of HP virulence factors (e.g., ureA, iceA2) in EBVaGC, implying that host-pathogen interactions may modulate tumor biology. Thus, whether the improved visibility observed post-eradication reflects mere resolution of inflammation or a genuine alteration of the tumor-host interface remains speculative. The “unmasking” observed here may represent only one facet of this complex interplay. Consequently, this strategy necessitates individualized decision-making based on lesion characteristics and bleeding risk.

### Limitations

Follow-up time is short, necessitating long-term monitoring. Although WLI showed no suspicious signs of submucosal invasion (e.g., fold fusion or non-extension sign) and definitive intramucosal carcinoma was confirmed by final pathology, we acknowledge that the omission of preoperative EUS is a limitation, because EUS can provide more direct evidence of tumor depth. PD-L1 expression was not assessed. Finally, as a single case report, these findings serve as clinical insights rather than universal conclusions.

## Conclusion

We report a rare case of synchronous intramucosal EBVaGC (GCLS type) managed successfully with ESD following short-term HP eradication. While this single case does not establish a causal link, it generates the hypothesis that pre-resection eradication might improve margin delineation in lesions obscured by active inflammation. Conversely, immediate ESD may be favored for distinct lesions. Furthermore, the detection of a “Lace Pattern” on ME-NBI or histology should raise suspicion for EBVaGC, warranting EBER testing for confirmation.

## Patient perspective

The patient expressed deep gratitude for the successful minimally invasive treatment and emphasized his hope that the detailed documentation of his case-particularly the role of H. pylori eradication in enhancing lesion visibility and the precision of endoscopic resection-would assist other clinicians in managing similar challenges more effectively. He reflected on the relief brought by the thorough diagnostic process and the expertise of the multidisciplinary team, which minimized discomfort and uncertainty. Looking back, he underscored the importance of advanced endoscopic techniques in achieving curative outcomes and hoped that his experience would contribute to earlier detection and improved care for patients with EBV-associated gastric lesions.

## Data Availability

The original contributions presented in the study are included in the article/supplementary material, further inquiries can be directed to the corresponding author.
